# Redox Metabolism During Aerial Exposure of the Sea Urchin *Echinometra lucunter*: An Ecophysiological Perspective

**DOI:** 10.3390/ani15091251

**Published:** 2025-04-29

**Authors:** Tatiana M. Pereira, Marina Minari, Juan Manuel Carvajalino-Fernández, Daniel C. Moreira, Marcelo Hermes-Lima

**Affiliations:** 1 Departamento de Biologia Celular, Universidade de Brasília, Brasilia 70910-900, Brazil; marinaminarirc@gmail.com (M.M.); 2 Departamento de Biología, Universidad Nacional de Colombia, Bogotá 111321, Colombia; jmcarvajalinof@unal.edu.co; 3 Faculdade de Medicina, Universidade de Brasília, Brasilia 70910-900, Brazil

**Keywords:** catalase, echinoderm, glutathione transferase, intertidal ecosystem, preparation for oxidative stress, redox balance, superoxide dismutase

## Abstract

Intertidal organisms experience daily environmental fluctuations, including changes in temperature, UV radiation, and aerial exposure during low tide, requiring physiological strategies for survival. One adaptation is the “preparation for oxidative stress” (POS), where antioxidants are upregulated preemptively to mitigate oxidative damage. While POS has been documented in over 120 species, in the case of intertidal species, most studies focus on single stressors under controlled-laboratory conditions. This study investigated POS in the sea urchin *Echinometra lucunter* under natural multi-stress conditions. Sampling occurred over a single day (from 5 a.m. to noon), analyzing three conditions: submerged (S), aerially exposed for 2 h (AE), and reimmersed for 2 h (R). There was a ~4 °C temperature increase in the AE group compared to the S group, with a peak of solar radiation during reoxygenation. Antioxidant enzyme activity—catalase, glutathione transferase (GST), and superoxide dismutase (SOD)—and lipid peroxidation (TBARS) were assessed in gonad and intestine tissues. GST activity increased by fourfold in the intestines of AE individuals, while TBARS was elevated in gonads, where no antioxidant upregulation happened. These results suggest that the POS-response (increase in GST) was triggered by the interaction of temperature and aerial exposure. This research emphasizes the critical importance of field studies in understanding complex, natural multi-stressor environments.

## Simple Summary

Animals living in coastal zones face daily environmental challenges, such as temperature changes, sunlight, and tidal fluctuations, during which sessile marine organisms are exposed to air for a few hours. These animals have developed behavioral, physiological, and biochemical adaptations to survive these conditions. Most studies examining biochemical responses simulate stressors in laboratory settings, focusing on a single factor. However, these results may not accurately reflect what occurs in nature. In this study, we analyzed the metabolism of free radicals and antioxidants in the sea urchin *Echinometra lucunter* under natural conditions, experiencing temperature and UV variations along with air exposure during low tide. Our findings reveal that *E. lucunter* activates its antioxidant system by increasing intestinal glutathione-S-transferase activity in response to the combined effects of temperature and air exposure, employing the “preparation for oxidative stress” strategy. This work emphasizes the need for field studies to truly understand how marine organisms respond to the challenges they face in their natural environments.

## 1. Introduction

Intertidal animals in coastal environments face daily exposure to multiple stressors associated with periodic tidal changes, including temperature fluctuations, intense UV radiation, and aerial exposure [1]. These stressors induce redox imbalance and oxidative stress, a state characterized by a limitation of antioxidant systems to control reactive species [2]. For example, temperature stress can increase reactive oxygen and nitrogen species (RONS) production and modulate antioxidant enzymes in intertidal organisms [3,4]. Similarly, exposure to UV radiation during low tide leads to redox imbalance and oxidative damage to proteins and membranes [5,6,7]. Furthermore, aerial exposure is a primary trigger of oxidative stress in intertidal zones, as these animals experience a daily tidal cycle with repeated periods of physiological hypoxia followed by reimmersion. Fluctuations in oxygen availability can increase mitochondrial ROS levels, potentially causing oxidative damage [8,9]. Consequently, the combined effects of temperature fluctuations, UV radiation, and aerial exposure are challenging for intertidal animals, potentially impacting their fitness and survival.

In many organisms, the biochemical mechanisms underlying the tolerance to such stressful situations include the upregulation of endogenous antioxidant systems. The precautionary increase in antioxidant levels, triggered by environmental stress, is a mechanism to mitigate potential oxidative stress in the subsequent phase. This mechanism, named “Preparation for Oxidative Stress” (POS), is a biochemical response that has been observed in animals under low oxygen stress and other stressors, such as high temperatures, freezing, hyposalinity, and UV radiation [2,10]. POS has been extensively observed in many animal species, including intertidal species [2]. By increasing the capacity of endogenous antioxidant systems, animals can mitigate the oxidative stress resulting from fluctuations in environmental factors, such as reoxygenation (e.g., reimmersion) after a period of hypoxia (e.g., aerial exposure, a condition of functional hypoxia) [11,12]. In the case of low oxygen stress, hypoxia-induced redox imbalance activates redox-sensitive transcription factors, such as Nrf2, which ultimately drive the overexpression of endogenous antioxidants [13].

Despite its prevalence across animal phyla, POS is still underexplored in aquatic ecosystems. This is evident in the case of marine invertebrates under entirely natural conditions, which, despite extensive examination in laboratory stress simulations, lack comprehensive field studies [12,14]. In ecophysiological research, the ability to observe the adaptive responses of organisms within their ecosystem, along with the dynamic interplay of simultaneous fluctuations of environmental factors, may reveal valuable insights under “real world” conditions. This can be achieved by natural experiments, in which animals are exposed to natural multi-stress scenarios. While laboratory experiments facilitate the elucidation of cause-and-effect relationships involving selected variables, animal responses may depend on the interactions among multiple stressors, and the outcomes may not always align with real-world scenarios [15]. Evidence from behavioral, physiological, and biochemical studies indicates that lab animals might respond differently from field animals due to the absence of simultaneous environmental fluctuations present in natural settings [16].

Over the past decade, there has been an increase in studies investigating the combined effects of multiple stressors in animals in an attempt to observe their true physiological responses [17]. Intertidal organisms have often been the subject of these investigations [18,19,20]. In this context, cross-tolerances may occur, and the physiological response of these organisms might depend upon the synergistic interactions between the abiotic factors [17]. Despite the increase in multi-stress studies on redox biology, few have been conducted in natural settings [21] and even fewer on marine invertebrates [5,14].

Sea urchins are key players in their ecosystems and a convenient model for studying biochemical adaptations to natural tidal stress [22]. Given the limited mobility of adults, they are especially susceptible to tidal variations, being trapped in ponds where environmental conditions (e.g., temperature, salinity, UV radiation, and oxygen availability) might reach challenging levels. The genus *Echinometra* is known for its resistance to environmental stress due to its low energy requirements and specialized rock-boring behavior [23]. This behavior enables individuals to carve protective cavities within rocky substrates, providing refuge from predators and reducing exposure to intense solar radiation and strong currents. Understanding how an *Echinometra* species responds to naturally dynamic stressors sheds light on their resilience mechanisms and offers essential insights into the cellular stress response of intertidal organisms.

In this work, we investigate the redox metabolism of the sea urchin *Echinometra lucunter* in response to a natural half-day tidal cycle. We explore whether this species presents the POS adaptive mechanism in the wild on a rocky reef beach located in western Brazil. The results are analyzed to determine how a multistressor environment might influence key biochemical variables, providing insights into the resilience mechanisms of intertidal organisms against natural challenges.

## 2. Materials and Methods

### 2.1. Animal Collection

**Figure 1:**
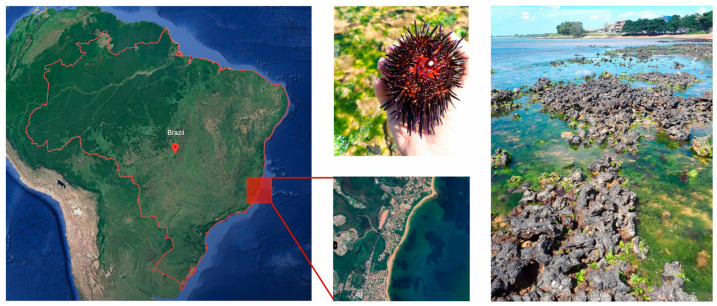
Sampling site of *Echinometra lucunter* on a rocky reef at Manguinhos Beach, Espírito Santo, Brazil (20°10′24.32″ S, 40°11′11.89″ W), located in the western Brazilian Atlantic Ocean.

Adult *Echinometra lucunter* specimens were collected at Manguinhos Beach, Espírito Santo, Brazil (20°10′24.32″ S, 40°11′11.89″ O). This beach’s intertidal zone is characterized by rocky reefs primarily composed of a laterite substrate with a moderate slope that becomes partially exposed during low tide. Geologically, these reefs are marked by ferruginous laterites due to extensive weathering on sedimentary substrates (Figure 1).

Sampling took place on 29 October (2020), a sunny day with clear sky, during the tidal cycle across the intertidal and inner sublittoral zones. The Submerged group (S; regarded as controls) (*n* = 7) was collected at 5 a.m. in the inner sublittoral zone, where specimens remained submerged. The Aerial Exposure group (AE) (*n* = 7) was collected in the intertidal zone at 7 a.m. when the tidal level was 0.0 m, and specimens had experienced 2 h of aerial exposure. The Reimmersion group (R) (*n* = 7) was collected from the same zone at 12 p.m., 2 h after the onset of high tide. After collection, sea urchins were promptly weighed, measured, and dissected to extract gonads and intestines, which were then frozen in liquid nitrogen and stored at −80 °C for further analysis.

The average weight and length of specimens in each group were as follows: (i) Submerged group: 176.41 ± 35.54 g and 43.0 ± 3.80 mm; (ii) Aerial Exposure group: 175.33 ± 36.92 g and 42.5 ± 4.83 mm; (iii) Reimmersion group: 169.79 ± 56.79 g and 43.5 ± 8.60 mm. This field research was conducted under licenses from ICMBio and SISBIO (permit number #61.407-1).

### 2.2. Meteorological Data

Meteorological data, including global solar radiation and air temperature, were obtained from the National Institute of Meteorology (INMET) using data from the A634 station, located approximately 40 km from Manguinhos Beach. Water temperature and dissolved oxygen levels were measured directly at the sampling site using a YSI Pro20 dissolved oxygen meter (YSI, Yellow Springs, OH, USA).

### 2.3. Activity of Antioxidant Enzymes

To determine antioxidant enzyme activity, frozen samples were weighed and diluted 1:10 (*w*/*v*) in a homogenization buffer, then manually homogenized on ice using a Tenbroeck-type glass tissue homogenizer. The buffer consisted of 20 mM Tris (pH 7.6), 1 mM EDTA, 0.5 mM sucrose, 0.15 mM KCl, 1 mM dithiothreitol (DTT), and 0.1 mM phenylmethylsulfonyl fluoride (PMSF). Homogenates were centrifuged at 10,000× *g* for 15 min at 4 °C, and the resulting supernatants were used immediately for antioxidant enzyme assays.

The activities of catalase (CAT), superoxide dismutase (SOD), and glutathione transferase (GST) were analyzed by spectrophotometry (SpectraMax 190, Molecular Devices, San Jose, CA, USA and Spectrophotometer SP-220, Biospectro, Curitiba, Brazil). Catalase activity was measured by monitoring the rate of H_2_O_2_ consumption, as previously described by Moreira et al. (2021) [12]. GST activity was assessed by quantifying the formation of a conjugate between 1-chloro-2,4-dinitrobenzene (CDNB) and reduced glutathione (GSH), as previously described [12]. SOD activity was determined using the xanthine oxidase assay [24]. Enzyme activities were expressed as units (U) per milligram of protein in the supernatant, with total protein concentration determined by the Bradford method [25].

### 2.4. Lipid Peroxidation Measurement

Lipid peroxidation levels were measured using a thiobarbituric acid reactive substances (TBARS) assay on frozen intestine and gonad samples. Samples were weighed and homogenized at a 1:20 (*w*/*v*) ratio on ice using a Tenbroeck-type glass tissue homogenizer in 20% (*w*/*v*) trichloroacetic acid (TCA). Following the protocol of Hermes-Lima and Storey (1995) [26], 400 µL of homogenate was combined with 200 µL of 7% (*w*/*v*) TCA, 0.75% (*w*/*v*) thiobarbituric acid (TBA), 50 mM sodium hydroxide (NaOH), and 0.1 µM butylated hydroxytoluene (BHT). Control aliquots were prepared under the same conditions, with TBA replaced by 3 mM HCl.

All samples were incubated at 98 °C for 20 min, then mixed and centrifuged at 2000× *g* for 5 min. Absorbance was measured at 532 and 600 nm using a SpectraMax 190 microplate reader (Molecular Devices).

### 2.5. Statistics

Statistical analyses were performed in R (version 4.4.3) within RStudio (version 2024.12.1.563; [27]). Prior to analysis, extreme outliers were identified and removed using the 3× IQR rule, where values beyond three times the interquartile range (IQR) from the first or third quartile were considered extreme. Normality was assessed using the Shapiro–Wilk test, and homogeneity of variances with Levene’s test. For normally distributed data with equal variances, one-way ANOVA was conducted, followed by Tukey’s HSD test for post hoc comparisons when significant differences were found (*p* < 0.05). For non-normally distributed data, a Kruskal–Wallis test was applied, with Dunn’s test and Bonferroni correction for multiple comparisons when necessary. Pairwise correlations between biochemical variables were calculated using Pearson’s correlation coefficient. Statistical significance was set at *p* < 0.05.

## 3. Results

**Figure 2:**
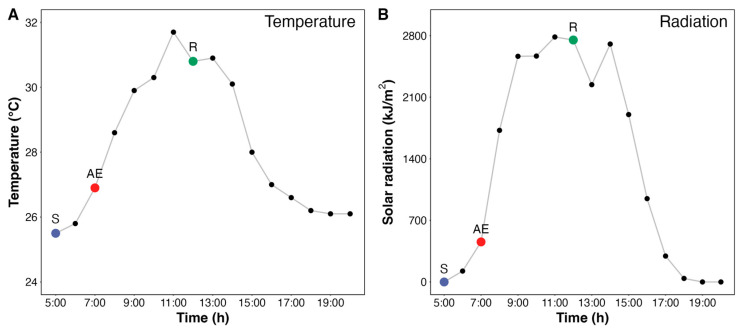
Temperature and solar radiation during the capture of adult sea urchin *Echinometra lucunter* specimens in a rocky reef at Manguinhos Beach, Brazil. (**A**) Temperature and (**B**) global solar radiation measured throughout the tidal cycle. Solid circles represent the time points where environmental data were recorded. Larger colored circles highlight the specific times when animals were collected for the experimental groups: blue for submersion (S), red for aerial exposure (AE), and green for reimmersion (R).

Environmental conditions during the sea urchin collection are summarized in Table 1. The air temperature was 25.5 °C at 5:00 a.m., reaching a maximum of 31.7 °C at 11:00 a.m. (Figure 2). During sampling, the air temperature was 27.8 °C (at 7:00 a.m.). Water temperature rose from 23.5 °C at 5:00 a.m. to 26.0 °C at noon. Solar radiation reached its maximum at 11:00 a.m., with 2784 kJ/m^2^/h. The cumulative solar radiation between 5:00 a.m. and 7:00 a.m. amounted to 579 kJ/m^2^ and between 7:00 a.m. and 12:00 p.m. amounted to 12,845 kJ/m^2^.

**Figure 3:**
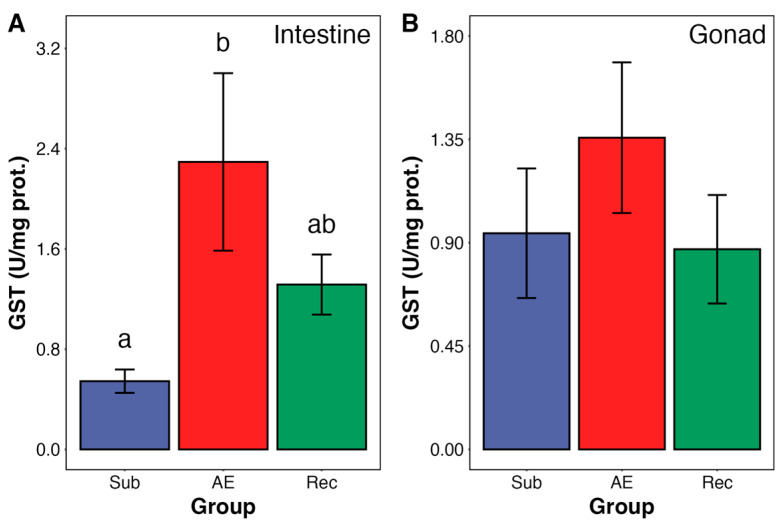
Glutathione transferase (GST) activity in the intestine (**A**) and gonad (**B**) of adult *Echinometra lucunter* during the natural tidal cycle under different conditions: submersion (blue), aerial exposure (red), and reimmersion (green). No significant differences were observed between groups in the gonad. In the intestine, GST activity was significantly higher in aerially exposed individuals compared to submerged ones (ANOVA, *p* = 0.039; Tukey *p* = 0.031). Groups not sharing the same letter are statistically different (*p* < 0.05). Data are presented as mean ± standard error (SE). N = 6–7 for both tissues.

The activity of GST in the intestine was approximately four times higher in air-exposed animals compared to those submerged (Figure 3A). During reimmersion, the activity was not different from controls (S group) or AE urchins (Figure 3A).

**Figure 4:**
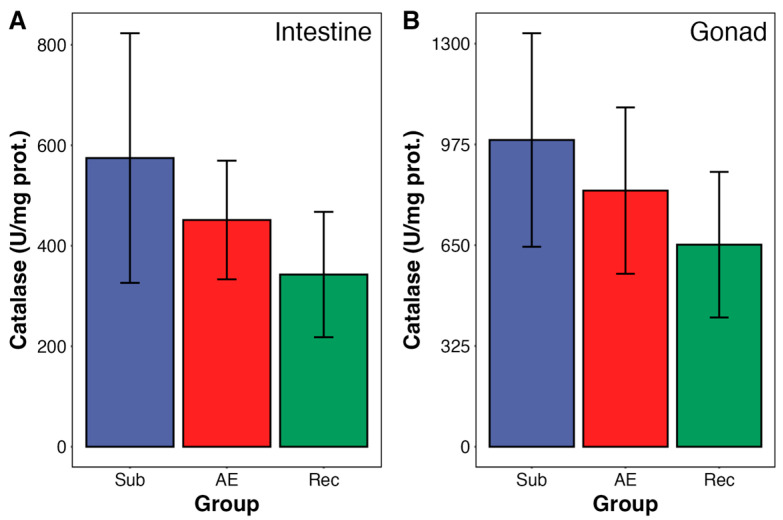
Catalase (CAT) activity in the intestine (**A**) and gonad (**B**) of adult *Echinometra lucunter* during the natural tidal cycle under different conditions: submersion (blue), aerial exposure (red), and reimmersion (green). No significant differences were observed between groups in both tissues. Data are presented as mean ± standard error (SE). N = 7 for both tissues.

**Figure 5:**
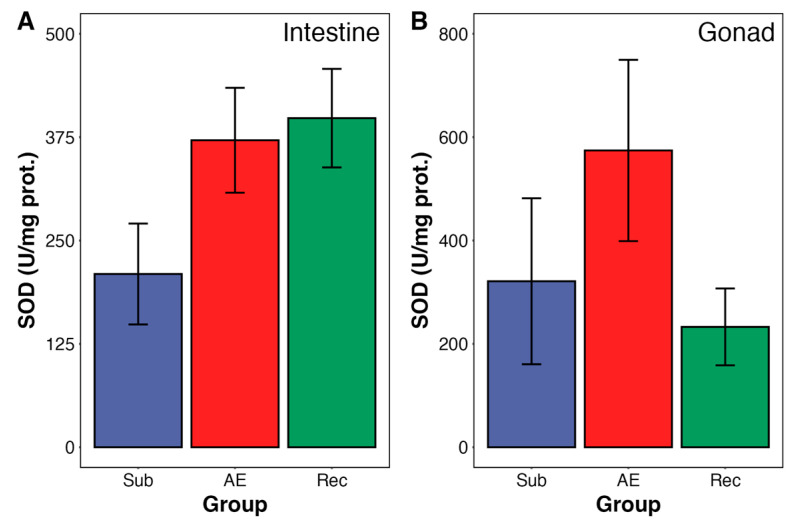
Superoxide dismutase (SOD) activity in the intestine (**A**) and gonad (**B**) of adult *Echinometra lucunter* during the natural tidal cycle under different conditions: submersion (blue), aerial exposure (red), and reimmersion (green). No significant differences were observed between groups in both tissues. Data are presented as mean ± standard error (SE). N = 4–5 for both tissues.

No changes were observed in gonadal GST activity during aerial exposure and re-submersion (Figure 3B). Moreover, catalase and SOD activities—from both organs—were unchanged during the tidal cycle in both urchin organs (Figure 4 and Figure 5). SOD from intestine presented a trend towards an increase in activity; however, it did not reach significance (*p* = 0.098).

**Figure 6:**
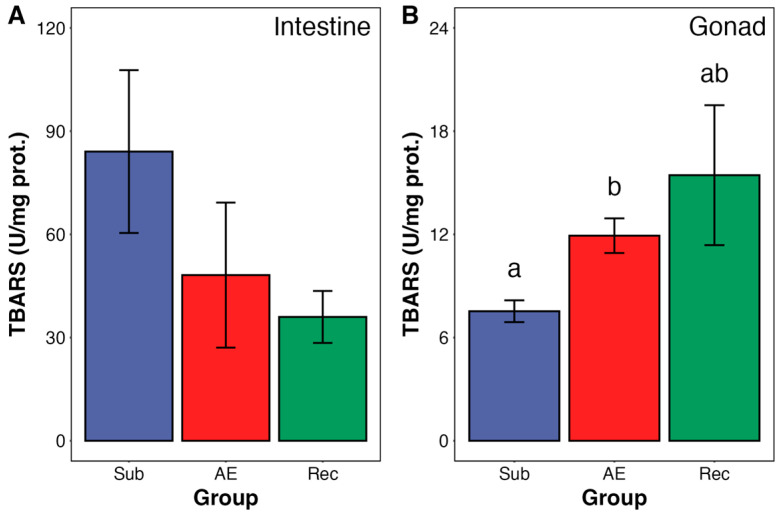
Lipid peroxidation levels (TBARS) in the intestine (**A**) and gonad (**B**) of adult *Echinometra lucunter* during the natural tidal cycle under different conditions: submersion (blue), aerial exposure (red), and reimmersion (green). In the gonads, TBARS levels were significantly higher in aerially exposed individuals compared to submerged ones (Kruskal–Wallis, *p* = 0.026; Dunn’s test *p* = 0.038). No significant differences were observed in the intestine. Groups not sharing the same letter are statistically different (*p* < 0.05). Data are presented as mean ± standard error (SE). N = 6–7 for both tissues.

Lipid peroxidation levels in gonads, determined as TBARS, were increased by 21% in aerial exposure. During reimmersion, the increase in lipid peroxidation was maintained, but it did not reach statistical significance (Figure 6). No change in TBARS was detected in the intestines.

Correlation analyses between variables in the same tissue were performed (six correlations per tissue). Only two correlations were statistically significant: SOD versus catalase in gonads (r = 0.621), and SOD versus GST in gonads (r = 0.659). Interesting GST in gonads correlated with GST in intestines (r = 0.803). All correlations are detailed in Supplemental Figure S1.

## 4. Discussion

Intertidal species have developed various adaptations and strategies to survive environmental stressors, including metabolic depression, osmoregulation, and production of photoprotective compounds (e.g., MAAs and pigments) [6,28,29,30]. Another strategy found in intertidal species is the “preparation for oxidative stress” (POS), whereby these animals upregulate their antioxidant system to manage the levels of reactive oxygen species (ROS) that may get increased under stress conditions, such as limitation of oxygen availability and higher temperatures [2,21]. In this study, we observed that the antioxidant enzyme GST was increased in the intestine of *E. lucunter* by about 300% during aerial exposure (AE), indicating the presence of the POS mechanism. Lipid peroxidation (as TBARS) was unchanged in the intestine, while it was modestly increased (by 21%) in gonads during AE, a tissue without activation of antioxidant capacity. This suggests that the increase in intestine GST was able to prevent a rise in TBARS.

Several mechanisms could underlie the increase in GST activity observed in the intestine of *Echinometra lucunter* during aerial exposure. One possibility is de novo protein synthesis, driven by the activation of redox-sensitive transcription factors such as Nrf2, which is known to regulate the expression of various antioxidant enzymes [31], including GSTs in aquatic invertebrates [32,33]. GSTs play crucial roles in antioxidant systems by catalyzing the conjugation of glutathione to electrophilic compound and detoxifying lipid peroxidation products, such as 4-hydroxynonenal [34]. Another possible mechanism is by decomposing hydroperoxides (which propagate the lipid peroxidation cascade) due to its peroxidase activity [35]. We have previously observed a strong upregulation of GST in insect larvae in diapause during the dry season, highlighting the role of GST in the response to environmental stressors [36].

The POS strategy has been demonstrated in over 120 animal species, from nine different phyla, including echinoderms. Most POS-related works on echinoderms were performed in holothurians (under heat stress and estivation; [37,38,39,40]) or in larvae of echinoids (under UV stress; [41]). Our work is the first to show that air exposure induces a POS response in the wild in an echinoderm species during the adult phase. However, other environmental factors might be contributing to the observed POS response in *E. lucunter*. The more suitable parameters are temperature and UV radiation, since both are increased from 5 a.m. to the end of the tidal cycle, at 12 p.m. It is well-known that increased temperatures may cause activation of antioxidant enzymes in several marine invertebrate species [20,42,43].

The effect of UV in activating antioxidant defenses was recently demonstrated to act in a hormetic way in small animals [10], including sea urchin larvae [41]. In our setting, temperature, UV radiation, and air exposure could also interact to induce the observed animal’s redox response. The effect of the interaction of stressors on the redox metabolism of different animals has been the subject of many studies in the past 10–15 years [20,44,45,46]. There are some examples of intertidal POS-positive species submitted to simulations of the interaction of stressors, including the mussel *Perna perna*, with air and diesel exposure, the oyster *Crassostrea hongkongensis* subjected to different hypoxia and salinity treatments, and the Manila clam *Ruditapes philippinarum* exposed to long-term aerial exposure combined with heat stress [18,19,47]. These studies demonstrate that the combination of stressors generates different metabolic responses compared to when only a single stressor is present, indicating cross-tolerances [17].

The current challenge for the POS theory is to investigate the impact of multi-stressors in nature, where environmental conditions fluctuate simultaneously. In other words, capturing the dynamics of natural multi-stress situations. Our study is one of the few that analyses intertidal animals in this context. The other two studies focus on the mussel *Brachidontes solisianus* and the gastropod *Littorina kurila* [5,12,14]. Moreira et al. (2023) demonstrated that when mussels are exposed to air receiving high incidence of solar radiation, they exhibit an upregulation of GSH levels accompanied by an increase in oxidative damage, indicating activation of the POS mechanism [5,12]. In contrast, no significant alterations in redox metabolism were observed when the mussels were exposed to aerial exposure under low solar radiation [5]. This study identified UV radiation and aerial exposure as primary stressors, considering that temperature was not a significant factor (it was stable during the tidal cycle). In the case of *L. kurila*, aerial exposure for 2 h induced an increase in TBARS; at 6 h under low tide catalase was increased, while TBARS returned to normal levels [14]. Since most air-exposure happened at night, UV might not have influenced the redox metabolism at low tide, nor temperature, since it was rather stable (about 22 °C for air temperature) [21].

Let’s analyze the putative action of temperature as a co-factor in triggering the biochemical responses in *E. lucunter* during the tidal cycle. Notably, species from the family Echinometridae are known for their resilience to environmental stress [23], and previous studies have shown that *E. lucunter* can tolerate temperatures up to 30 °C without significantly affecting fertilization, embryonic development, immune function, or mortality [48,49,50,51]. In this study, the environmental temperature varied from 23.5 °C (in underwater controls at 5:00 a.m.) to 27.8 °C (in AE at 7:00 a.m.) and 26°C (reimmersion at noon). This is a fluctuation of 4.3 °C, considering water temperature in underwater and reimmersion groups and air temperature for the AE group (see Table 1). Similarly, a study analyzing the sea urchin *Arbacia punctulata* at three temperatures, 24 °C (control), 28 °C, and 32 °C, found a significant increase in carbonyl proteins in the 28 °C and 32 °C groups, with the highest level of HSP70 observed at 32 °C [3]. In contrast, Sherman (2015) [52] demonstrated that *E. lucunter* exhibits seasonal acclimation to temperature changes, with a thermal safety margin for physiological adaptation of 3.0 to 3.6 °C. Considering this information, it is possible that the redox metabolism of *E. lucunter* could have been influenced by the 4.3 °C increase in temperature during aerial exposure. Moreover, this could have acted in synergy with the effects of functional hypoxia caused by aerial exposure.

On the other hand, exposure to UV probably did not play a relevant role in the redox metabolism of *E. lucunter* during aerial exposure. This is because the accumulation in solar radiation between 5:00 and 7:00 a.m. was quite low: only 579 kJ/m^2^. In addition, the activity of GST presented a trend to decrease from AE to reimmersion (even though was not significant), when solar radiation summed over 12,000 kJ/m^2^. The POS-response linked to air-exposure in mussels *B. solisianus* was only observed after exposure to ~15,000 kJ/m^2^ of total solar radiation [5]; ~5500 kJ/m ^2^ failed to induce POS in this mollusk.

Even though we cannot fully disentangle the effects of temperature and aerial exposure as triggers of the POS response, our findings suggest that temperature alone was unlikely to be the primary driver. While animals in the AE group experienced both air exposure and temperature changes, they were also subjected to further temperature fluctuation after reimmersion. From aerial exposure (7 a.m.) to reimmersion (noon), temperature continued to rise—increasing from 27.8 °C to 30.8 °C in air and from 24.0 °C to 26.0 °C in water. Despite this additional increase in temperature, no significant changes were observed in redox biomarkers during this transition. This contrasts with the AE group, where significant changes in redox metabolism biomarkers were observed. If temperature had been the main factor triggering POS, we would have expected a progressive increase in antioxidant responses as temperature continued to rise, which was not the case.

The relatively moderate temperature fluctuations observed in our study (a maximum increase of 4.3 °C) are unlikely to have been sufficient to independently drive a strong redox response in *E. lucunter*. Instead, the most pronounced redox response—the fourfold increase in intestinal GST activity—occurred during aerial exposure, when the sea urchins experienced functional hypoxia due to air exposure, rather than during the later period when the temperature was at its highest. This pattern strongly suggests that aerial exposure, rather than temperature, was the primary driver of the observed POS response. While we cannot rule out potential synergistic effects between temperature and hypoxia, as both factors often interact in intertidal species, the absence of further biochemical changes during reimmersion supports the conclusion that aerial exposure played a dominant role in triggering POS in *E. lucunter* under the natural tidal cycle.

While this study advances our understanding of the biochemical responses of *Echinometra lucunter* to air exposure, some limitations should be considered. First, conducting the experiment on a day with lower solar radiation (e.g., a cloudy winter day) could help clarify the influence of temperature and UV exposure in triggering the observed biochemical responses. Replicating the natural experiment under a similar tide cycle in a cloudy day with lesser temperature variations (from morning to noon) could help verify our proposal for the possible effect of temperature on POS induced by air exposure. Additionally, the lack of measurements for other players in redox metabolism, such as glutathione peroxidase, glutathione, and protein carbonyls, limits our ability to fully characterize the antioxidant response and oxidative damage. Including these markers could provide a more comprehensive picture of redox dynamics. Despite these limitations, the robust increase in GST activity—a fourfold change—strongly supports the occurrence of the POS mechanism in *E. lucunter*, reinforcing its physiological relevance in intertidal environments. Future studies incorporating additional oxidative stress markers and varying environmental conditions will help refine our understanding of POS in marine invertebrates.

## 5. Conclusions

This study provides evidence that *Echinometra lucunter* employs the POS mechanism during aerial exposure in its natural intertidal habitat, as indicated by the marked increase in intestine GST activity. While environmental stressors such as temperature fluctuations, UV radiation, and functional hypoxia likely interact to shape the redox response, our findings suggest that temperature and, mainly, aerial exposure were main drivers of antioxidant activation. The absence of oxidative damage in the intestine, despite increased lipid peroxidation in the gonads, further supports the protective role of GST in mitigating oxidative stress. Although more biochemical markers and studies under varying environmental conditions are needed to refine our understanding of POS in intertidal echinoderms, these results underscore the importance of field-based ecophysiological research to capture the complexity of natural multistressor environments.

## Figures and Tables

**Table 1:** Environmental conditions to which adult sea urchin, *Echinometra lucunter*, were exposed during the natural tidal cycle while subjected to submersion (S, at 5:00 a.m.), air exposure (AE, at 7:00 a.m.), or reimmersion (R, at 12:00 p.m.) in a rocky reef in the western Brazilian Atlantic Ocean. The solar radiation values represent the cumulative solar radiation from when each group was collected.

Condition	Experimental Group		
	S	AE	R
Time (h)	5	7	12
Tide (m)	0.1	0.0	1.3
Solar radiation (kJ/m^2^)	0.0	2302.2	15,210.9
Air temperature (°C)	25.5	27.9	30.8
Water temperature (°C)	23.5	24.0	26.0
Dissolved oxygen (DO mg/L)	7.8	–	6.1
Relative DO (%)	112	–	92

## Data Availability

The original contributions presented in this study are included in the article/Supplementary Material. Further inquiries can be directed to the corresponding author.

## Supplementary Materials

The following supporting information can be downloaded at: https://www.mdpi.com/article/10.3390/ani15091251/s1, Figure S1: Correlation Matrix and Pairwise Relationships of Biochemical Variables in *Echinometra lucunter* Tissues.
