# Supplementary figures and images for: Redox Metabolism During Aerial Exposure of the Sea Urchin Echinometra lucunter: An Ecophysiological Perspective
Source: Animals (Basel). 2025 Apr 29;15(9):1251. doi: 10.3390/ani15091251 (PMC12070949; doi:10.3390/ani15091251)

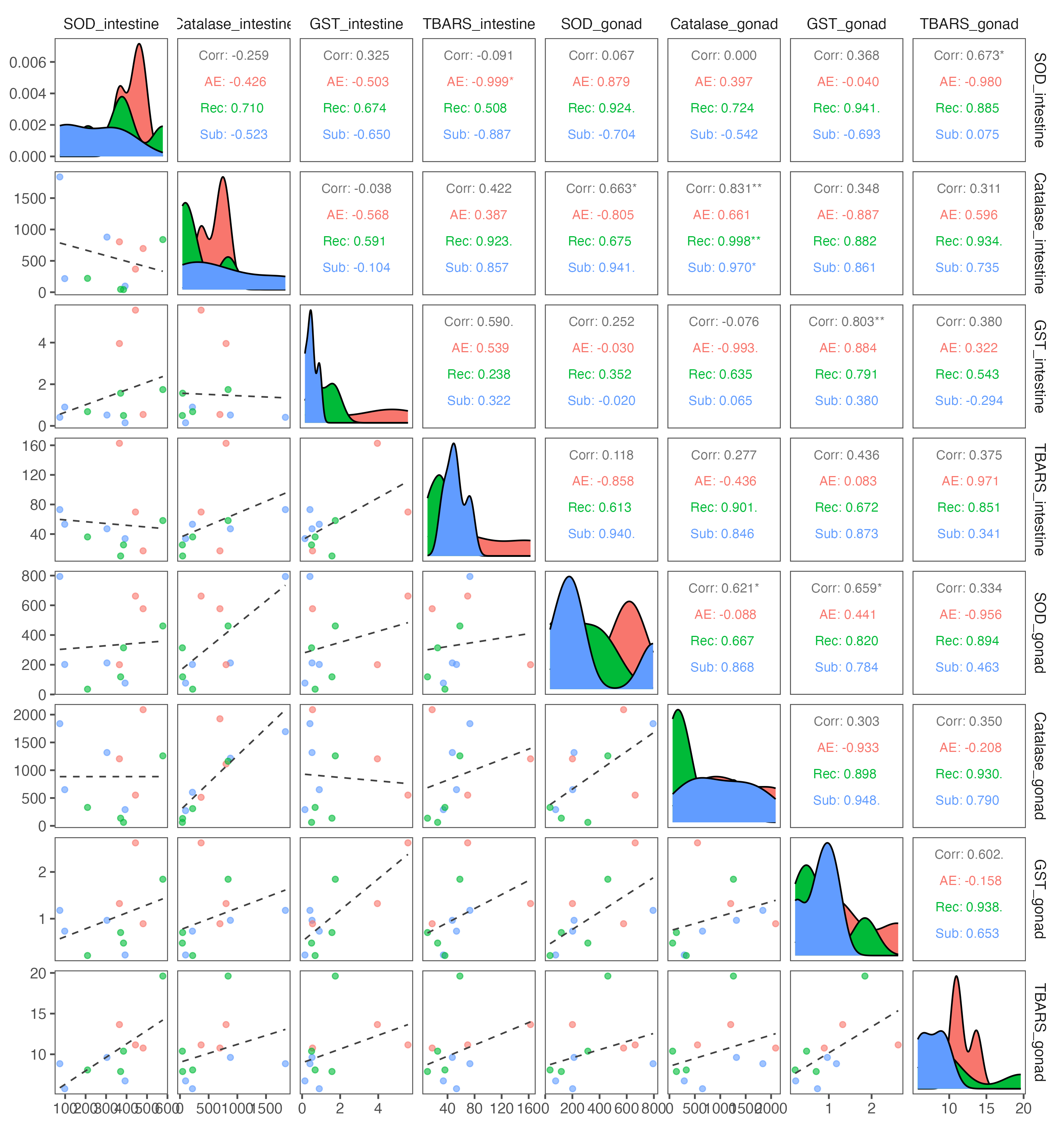

Supplement: Supplementary file 1 [file animals-15-01251-s001.zip › animals-3554109-supplementary Figure S1.png]
